# Succinate aggravates intestinal injury in mice with necrotizing enterocolitis

**DOI:** 10.3389/fcimb.2022.1064462

**Published:** 2022-11-28

**Authors:** Xiao-Lin Yan, Xiao-Chen Liu, Yu-Ni Zhang, Ting-Ting Du, Qing Ai, Xiong Gao, Jing-Li Yang, Lei Bao, Lu-Quan Li

**Affiliations:** Department of Neonatology Children’s Hospital of Chongqing Medical University, National Clinical Research Center for Child Health and Disorders, Ministry of Education Key Laboratory of Child Development and Disorders, Chongqing Key Laboratory of Pediatrics, Chongqing, China

**Keywords:** necrotizing enterocolitis, succinate, metabolites, gut microbiota, intestinal inflammation

## Abstract

**Background:**

Necrotizing enterocolitis (NEC) is the most prevalent gastrointestinal disorder that predominantly threatens preterm newborns. Succinate is an emerging metabolic signaling molecule that was recently studied in relation to the regulation of intestinal immunity and homeostasis. We aimed to investigate the relationship between NEC and gut luminal succinate and preliminarily explored the effect of succinate on NEC pathogenesis.

**Methods:**

Fecal samples from human neonates and mouse pups were analyzed by HPLC – MS/MS and 16S rRNA gene sequencing. C57BL/6 mice were randomly divided into four groups: control, NEC, Lsuc, and Hsuc. The mortality, weight gain, and intestinal pathological changes in four mouse groups were observed. Inflammatory cytokines and markers of macrophages were identified by quantitative real-time PCR. Succinate receptor 1 (SUCNR1) localization was visualized by immunohistochemistry. The protein levels of SUCNR1 and hypoxia-inducible factor 1a (HIF-1a) were quantified by western blotting.

**Results:**

The levels of succinate in feces from NEC patients were higher than those in feces from non-NEC patients (*P <*0.05). In the murine models, succinate levels in intestinal content samples were also higher in the NEC group than in the control group (*P <*0.05). The change in succinate level was closely related to intestinal flora composition. In samples from human neonates, relative to the control group, the NEC group showed a higher abundance of *Enterobacteriaceae* and a lower abundance of *Lactobacillaceae* and *Lactobacillus (P <0.05)*. In the murine models, relative to the control group, increased abundance was observed for *Clostridiaceae*, *Enterococcaceae*, *Clostridium_sensu_stricto_1*, and *Enterococcus*, whereas decreased abundance was observed for *Lactobacillaceae* and *Lactobacillus (P <0.05)*. Increased succinate levels prevented mice from gaining weight, damaged their intestines, and increased their mortality; upregulated the gene expression of interleukin-1β (IL-1β), IL-6, IL-18 and tumor necrosis factor (TNF); and downregulated the gene expression of IL-10 and transforming growth factor (TGF)-β. Exogenous succinic acid increased inducible nitric oxide synthase (iNOS) gene expression but decreased Arginase-1 (Arg1) gene expression; and increased the protein expression of SUCNR1 and HIF-1a.

**Conclusion:**

Succinate plays an important role in the development of necrotizing enterocolitis severity, and the activation of the HIF-1a signaling pathway may lead to disease progression.

## Introduction

Necrotizing enterocolitis (NEC) is a severe gastrointestinal disorder and is the most common such disorder that predominantly affects premature infants, resulting in high morbidity and mortality (Flahive et al., 2020). An estimated 2%-5% of neonates in neonatal intensive care units have diagnosed NEC, and the incidence of extremely preterm newborns is approximately 8.9% (Jiang et al., 2020; Bell et al., 2022). The mortality rate of confirmed NEC (Bell stage 2a+) ranges from 15% to 30% and is even higher in infants who require surgical intervention (Flahive et al., 2020; Blakely et al., 2021). Furthermore, surviving patients may have substantial long-term neurodevelopmental and growth sequelae (Chen et al., 2021; Imren et al., 2022). Despite decades of research, the pathogenesis and treatment of NEC remain unclear. Therefore, the mechanisms of pathogenesis need to be further elucidated to identify new possibilities for treating NEC to reduce its morbidity and improve its prognosis.

Although the pathogenesis of NEC is complex and multifactorial, large amounts of evidence have reported that gut microbiota dysbiosis plays an essential role in NEC occurrence and progression (Claud and Walker, 2001; Tarracchini et al., 2021). Previous studies revealed that neonates with NEC have significantly different gut microbiomes than non-NEC neonates (Liu et al., 2022b): the diversity of bacteria is reduced, and the abundance of *Enterobacteriaceae* is increased in neonates with NEC, which can activate TLR4 and promote NEC progression (Shaw et al., 2021). Additionally, with the change in the gut microbiota, metabolites show corresponding changes; the affected metabolites are generally considered to act as a bridge between flora and host communication and may also be involved in the pathogenesis of NEC (Krautkramer et al., 2021; Zhou et al., 2022a). For example, several studies have demonstrated the effect of short-chain fatty acids on the progression of intestinal health in patients with NEC and demonstrated that they show predictive value for NEC development (Roy et al., 2018; Sun et al., 2021; Liu et al., 2022a).

Succinate, widely regarded as an intermediate molecule of the mitochondrial tricarboxylic acid cycle, has recently emerged as a key player in maintaining intestinal homeostasis, intestinal energy metabolism, and immune regulation (De Vadder et al., 2016; Banerjee et al., 2020; Zhou et al., 2022b). A study pointed out that succinate can alter bacterial virulence while increasing bacterial invasion in inflammatory bowel disease (Zaidi et al., 2020). Together, these multibranched literature pointed out that succinate is not a simple intermediate metabolite, but act as a multifaceted signal molecule to activate various downstream factors, thus participating in the response to intestinal injury (Connors et al., 2018). However, currently succinate has not yet been studied in NEC as a multifunctional metabolite. In this study, we aimed to explore the relationship between succinate levels and NEC and to preliminarily reveal the mechanism of succinate involvement in the pathogenesis of experimental NEC models.

## Materials and methods

This study involving human participants was reviewed and approved by the Ethics Committee of Children’s Hospital of Chongqing Medical University (No. 2021.23). The patients of the enrolled neonates all signed informed consent forms. Our animal study was approved by the Animal Ethics Committee at Chongqing Medical University (No. CHCMU-IACUC20210316004).

### Participants and sample collection

Neonates with NEC and control neonates from the Department of Neonatology at the Children’s Hospital of Chongqing Medical University were recruited in this study, and fecal samples collection took place between April 2021 and November 2021.Twelve preterm neonates (gestational age<37weeks) with confirmed NEC (Bell stage 2a+) were included in the NEC group, and non-NEC newborns matched to NEC newborns by gestational age, birth weight, mode of delivery, day of sample collection, neonatal feeding type and antibiotic exposure were considered the control group.

Fecal samples from NEC newborns were obtained with disposable sterile swabs on the day of diagnosis of NEC; additionally, the control samples were collected at the same day age as their matched NEC cases. Freshly evacuated feces were transferred to sterile tubes and immediately stored at – 80°C for subsequent metabolite identification and flora classification.

### Quantification of succinate in samples using HPLC – MS/MS analysis

Sixty milligrams of each thawed fecal sample were transferred to an EP tube, after which 1 mL of cold methanol/acetonitrile/H2O (2:2:1, v/v/v) was added, and the mixture was adequately vortexed. The lysate was homogenized twice with an MP homogenizer (24×2, 6.0M/S, 60s). The homogenate was sonicated at low temperature (30 min/once, twice), the mixture was centrifuged for 20 min (14000 g, 4°C), and the supernatant was collected and lyophilized in a vacuum centrifuge. For the next LC – MS/MS analysis, the samples were redissolved in 100 μl acetonitrile/water (1:1, v/v) and adequately vortexed, centrifuged (14000 g, 4 °C, 15min) and the supernatant was collected. The sample extracts were analyzed using UHPLC (1290 Infinity LC, Agilent Technologies). Mobile phase contained A=10 mM CH3COONH4 in water and B= acetonitrile. The samples were placed in the automatic sampler at 4 °C, and the column temperature was kept constant at 45 °C, The flow rate of the gradient was 300, ul/min, and a 2 µL aliquot of each sample was injected. The gradient was as follows: 90% B was linearly reduced to 40% B over 0-18min, followed by an increase to 90% B over 0.1 min, which was maintained for 18.1-23min. QC samples prepared from the pooled samples, were added to the column at regular intervals in the analysis sequence (one QC after every 5 samples) to monitor the precision and stability of the method during its operation. Mass spectrometry was performed using QTRAP (AB Sciex 5500). In ESI negative mode, the conditions were set as follows: source temperature 450°C, ion source gas1(Gas1): 45, ion source gas2(Gas2): 45, curtain gas (CUR):30, ionspray voltage floating(ISVF)-4500 V; and adoption of the MRM-mode detection ion pair. Data processing: Data acquisition and processing were accomplished using Multiquant software. The retention time was corrected based on the standard of each energy metabolite, and the metabolites were identified.

### Fecal sample microbiome sequencing

Microbial DNA was extracted from fecal samples using a Magnetic Soil and Stool DNA Kit (TIANGEN). The DNA concentration and purity were monitored on 1% agarose gels. The V3-V4 region of the microbial 16S rRNA genes was amplified using the 341F-806R primer set:341F (5’- CCTAYGGGRBGCASCAG -3’) and 806R (5’- GGACTACNNGGGTATCTAAT -3’). The PCR products were mixed with the same volume of 1X loading buffer (containing SYBR green), and electrophoresis was performed on a 2% agarose gel for detection. Further experiments were conducted on samples with bright main bands between 400 and 450 bp. Then, PCR products were purified with a Qiagen Gel Extraction Kit (Qiagen). Sequencing libraries were pooled using a TruSeq^®^ DNA PCR-Free Sample Preparation Kit (Illumina) and added the index codes. The Qubit@ 2.0 Fluorometer (Thermo Scientific) and Agilent Bioanalyzer 2100 were used to assess the quality of the library. Finally, the Illumina NovaSeq6000 platform was used to sequence the library, resulting in paired-end reads of 250 bp. The raw FASTQ data were quality-filtered by fastp version 0.19.6 and then merged by FLASH version 1.2.7 with the following criteria: reads containing ambiguous characters or that could not be assembled were discarded; reads with a quality score of less than 20 were truncated; and sequences with a length greater than 10 bp were overlapped. The maximum mismatch ratio of the overlap region is 0.2. Based on 97% sequence similarity, the optimized sequences were clustered into operational taxonomic units (OTUs) using UPARSE 7.1 (Edgar, 2013). The number of 16S rRNA gene sequences from each sample was rarefied to the minimum number of sample sequence, which still yielded an average Good’s coverage of 99.09%.

### Animal model and experimental design

C57BL/6J wild-type (WT) mice were originally purchased from the Animal Experiment Center of Chongqing Medical University and maintained by the Animal Research Platform of Children’s Hospital Affiliated with Chongqing Medical University. Both male and female of mouse pups were used, along with littermate controls when possible. On the 10th day after birth, newborn mice (3-5 g) were randomly divided into four groups (n=20 per group): control, NEC, Lsuc (mice with NEC that received 50mM succinic acid intervention), and Hsuc groups (mice with NEC that received 100mM succinic acid intervention).

NEC induction was performed as previously described (Ji et al., 2021). Breastfed control mice were cohoused with their mothers and did not receive any intervention before being humanely killed. The NEC group was hand-fed formula milk (2 g of Esbilac Puppy Milk and 3.33 g of Similac Advance Replacer in 10 ml drinking water), and for the succinic acid intervention groups, mice were given formula milk containing succinic acid at 50 mM or 100 mM (2 g of Esbilac Puppy Milk and 3.33 g of Similac Advance Replacer in 10 ml succinic acid water at a 50 mM or 100 mM concentration, adjusted to pH 6.5-7.5 using NaOH to match drinking water), which was administered at 30 ul/g body weight *via* oral gavage every 4 h for 4 days. These mice were also subjected to hypoxia (100% nitrogen gas for 90 s) followed by cold stress (placement in a refrigerator at 4°C for 10 min) twice a day. Before the first feeding every morning, each mouse from the four groups was weighed using a weighing scale and assessed visually for their response to tail stimulation. At the 96-hour experimental endpoint, mice were killed humanely, and the intestinal contents and tissues were collected carefully and stored at -80 degrees in a freezer for subsequent experiments. Microbial composition analysis of intestinal contents and quantitative analysis of succinate were conducted as described above.

### Histological scoring

Murine terminal ileal tissues (1 cm each) were fixed overnight in 4% paraformaldehyde and then embedded in paraffin. Subsequently, 4 μm sections were stained with hematoxylin-eosin (HE), and histopathological analysis was performed in blind by a pathologist based on a published NEC damage scoring system (Halpern et al., 2006). Histologic changes were graded as follows: 0 (normal), no damage; 1 (mild), slight submucosal and/or lamina propria separation; 2 (moderate), moderate separation of submucosa and/or lamina propria, and/or edema in submucosal and muscular layers; 3 (severe), severe separation of submucosa and/or lamina propria, and/or severe edema in submucosa and muscular layers, region villous sloughing; 4 (necrosis), loss of villi and necrosis.

### Immunohistochemistry

After deparaffinization and rehydration, the tissue sections were placed in a microwave oven in citric acid (PH 6.0) antigen retrieval buffer and heated for 25 minutes for antigen retrieval. Endogenous peroxidases were inhibited, and nonspecific binding was blocked with 3% bovine serum albumin (BSA) for 30 min. The slides were washed with phosphate buffer saline (PBS) and incubated at room temperature for 50 minutes with the horseradish peroxidase (HRP)-conjugated secondary antibody following overnight incubation with rabbit anti-GPR91 (ab272856) at a 1:200 dilution. After diaminobenzidine (DAB) coloration, sections were counterstained for approximately 3 minutes with hematoxylin stain solution, dehydrated, and covered with cover slips. Images were obtained using a microscope (Image Viewer G, China). The nucleus is stained blue by hematoxylin, and positive DAB expression is brownish yellow.

### Quantitative real-time PCR

Total RNA was obtained from the distal ileum using an RNAiso Plus kit (Takara, Japan). For cDNA synthesis, total RNA was reverse transcribed to cDNA with a PrimeScript RT reagent kit with gDNA Eraser (Takara, Japan) following the kit instructions. SYBR green-based RT – qPCR was performed on a Bio-Rad CFX96 system using a TB Green Premix Ex Taq II Kit (Takara, Japan). The stably expressed housekeeping gene hypoxanthine phosphoribosyltransferase 1 (Hprt1) was used to normalize mRNA expression, and relative expression was quantified using the ΔΔCT method in Microsoft Excel (Microsoft, USA). The specified primers were designed using the NCBI Primer-BLAST tool (https://www.ncbi.nlm.nih.gov/tools/primer-blast/) or available from previous literature (Macias-Ceja et al., 2019; Wu et al., 2020; Mills et al., 2021; Sun et al., 2021) and Primer Bank (http://pga.mgh.harvard.edu/primerbank/index.html), and purchased from Sangon Company (Shanghai, China) with the detailed sequence information provided in **Table 1**.

**Table 1:** Primer sequences.

Primer	Direction	Sequence	Reference or source
Hprt1	Forward	TCAGTCAACGGGGGACATAAA	NCBI Primer-BLAST
	Reverse	GGGGCTGTACTGCTTAACCAG	
IL-6	Forward	GAGTCCTTCAGAGAGATACAGAAAC	Macias-Ceja et al., 2019
	Reverse	TGGTCTTGGTCCTTAGCCAC	
IL-18	Forward	GACTCTTGCGTCAACTTCAAGG	Primer Bank
	Reverse	CAGGCTGTCTTTTGTCAACGA	
TNF	Forward	CCTGTAGCCCACGTCGTAG	Primer Bank
	Reverse	GGGAGTAGACAAGGTACAACCC	
IL-10	Forward	GGACAACATACTGCTAACCGAC	Macias-Ceja et al., 2019
	Reverse	CCTGGGGCATCACTTCTACC	
TGF-β	Forward	GCCTGAGTGGCTGTCTTTTG	NCBI Primer-BLAST
	Reverse	GCCCTGTATTCCGTCTCCTT	
IL-1β	Forward	TGGTGTGTGACGTTCCCATT	Sun et al., 2021
	Reverse	CAGCACGAGGCTTTTTTGTTG	
iNOS	Forward	CCAAGCCCTCACCTACTTCC	Mills et al., 2021
	Reverse	CTCTGAGGGCTGACACAAGG	
Arg1	Forward	CCACAGTCTGGCAGTTGGAAG	Wu et al., 2020
	Reverse	GGTTGTCAGGGGAGTGTTGATG	

### Protein extraction and western blotting

Total protein was extracted using RIPA buffer (Beyotime, China) supplemented with a protease inhibitor cocktail for mammalian cell and tissue extracts (Beyotime, China), homogenized using a precooled electric homogenizer for 5 min, and incubated on ice for 20 min. Lysates were centrifuged at 14,000 rpm for 5 min at 4°C, and the supernatants were obtained. Total protein was quantified using an enhanced BCA protein assay kit (Beyotime, China). For protein analysis, we mixed the protein samples with loading buffer in a certain proportion and boiled for them 5 minutes. Protein samples were separated using 10% SDS – PAGE and then transferred onto polyvinylidene difluoride (PVDF) membranes (Millipore). Membranes were washed and blocked using NcmBlot blocking buffer (NCM Biotech, China) for 15 min at room temperature and then incubated with anti-ACTIN(HRP-conjugate) (700068, ZENBIO, 1:10000), anti-GPR91 (ab272856, 1:1000) and anti-HIF1A (AF1009, 1:1000) antibodies overnight at 4°C. After washing of the membranes, membranes were incubated for 1 h with the HRP-conjugated secondary antibodies. Then, the western blot results were visualized using enhanced chemiluminescence (ECL, ZENBIO Biotechnology, China). Quantification of the bands was done with ImageJ and normalized to beta-actin measured on the same membranes.

### Statistical analysis

All data were analyzed and graphed using GraphPad Prism 8.0 or SPSS statistical software (version 25; Chicago, IL, United States). The paired t test, t test, Wilcoxon matched-pairs signed rank test or Mann–Whitney rank-sum test was used as appropriate. For multigroup analysis, the Kruskal – Wallis test or one-way analysis of variance (ANOVA) with Dunn’s *post hoc* test was used. Survival was analyzed with log rank test. Proportions were compared using the Chi-square test or Fisher’s exact test. R (version 3.3.1) was used to generate a correlation heatmap based on the Spearman correlations between energy metabolite concentrations and the microbiota composition. Data are represented as mean ± standard deviations (SD) or median (interquartile range [IQR]). All statistical tests were two-sided and were performed at a significance level of *P <*0.05.

## Results

### Patient characteristics

A total of 12 NEC newborns and 12 matched non-NEC control newborns were enrolled in this study. The baseline clinical characteristics of the enrolled NEC patients and controls are summarized in **Table 2**. The two groups did not differ significantly in terms of baseline characteristics, including gestational age, mode of delivery, type of feeding, antibiotic exposure at the point of sample collection and birth weight (*P*>0.05).

**Table 2:** Patient characteristics.

Variables	Control (n=12)	NEC (n=12)	*χ^2^/Z/t*	*P* value
Gestational age, ¯x ± SD, w	31.7 ± 2.32	31.33 ± 2.1	-0.409	0.687
Birth weight, ¯x ± SD, g	1720.83 ± 541.32	1577.08 ± 575.26	-0.63	0.535
Female, % (n)	33.3(4)	25(3)	/	1.000
Vaginal delivery, %(n)	41.7(5)	33.3(4)	/	1.000
Intrauterine distress, %(n)	25(3)	8.3(1)	/	0.590
Apgar 1 min, M (IQR)	8(6.25-9)	8.5(7.25-9)	-0.521	0.630
Age at enrollment, M (IQR), d	7(3-16.5)	17(9.25-31.5)	-1.937	0.052
CGA at enrollment, ¯x ± SD, w	33.43 ± 2.36	34.36 ± 2.09	1.021	0.318
Breast feeding, %(n)	58.3(7)	41.7(5)	/	0.684
Antibiotic exposure during sample collection, %(n)	41.7(5)	41.7(5)	/	1.000
PROM, %(n)	25(3)	33.3(4)	/	1.000
Chorioamnionitis, %(n)	8.3(1)	8.3(1)	/	1.000
GDM, % (n)	25(3)	41.7(5)	/	0.667
Surgical treatment, %(n)	0.0(0)	33.3(4)	/	0.093

NEC, necrotizing enterocolitis; IQR, interquartile range; CGA, corrected gestational age; PROM, premature rupture of membranes>18h;GDM, gestational diabetes mellitus.

### Succinate measurement and analysis of the relationship between succinate and the gut microbiota

**Figure 1:**
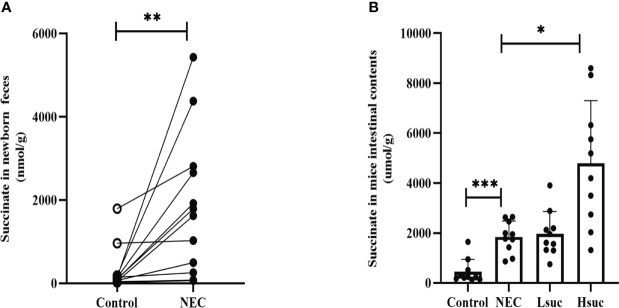
The levels of intestinal succinate were increased in both human neonates and mouse pups with NEC. Succinate levels **(A)** in feces from control and NEC human neonates (n=12 per group), and **(B)** in mouse intestinal contents from four independent groups (n=10 per group). Statistical analysis was performed with paired t-test or one-way ANOVA followed by Dunnett’s posttest. Bars in the graph represent the mean ± SD, and significant differences are shown by *P <0.05, **P <0.01, ***P <0.001. NEC, necrotizing enterocolitis; Lsuc, mice with NEC receiving 50 mM succinic acid intervention; Hsuc, mice with NEC receiving 100 mM succinic acid intervention.

To evaluate succinate levels among neonates with NEC, we compared the difference in fecal succinate levels between neonates with NEC and those without NEC. The levels of succinate in feces from NEC human neonates were markedly higher than those from non-NEC human neonates **(** **Figure 1A** **)**. Additionally, NEC and succinic acid-intervened mice presented significantly higher succinate concentrations than control mice **(** **Figure 1B** **)**.

**Figure 2:**
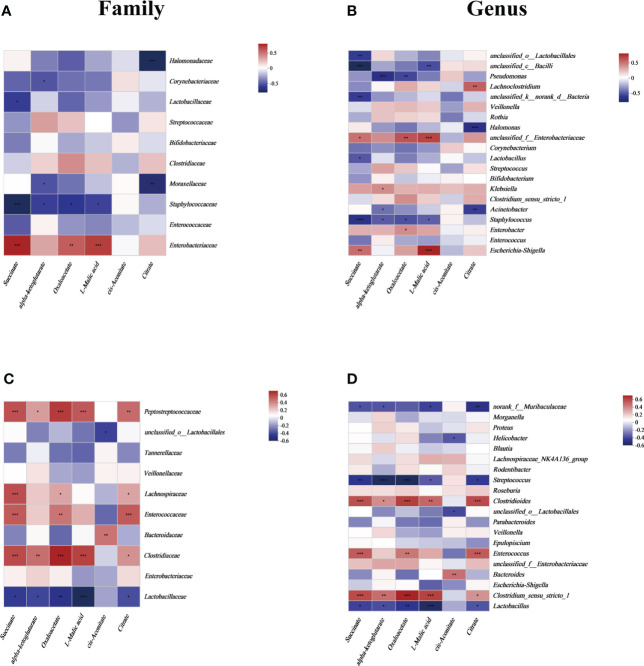
The relationship between the gut microbiota and energy metabolites at the **(A)** family level and **(B)** genus level in human neonates and at the **(C)** family level and **(D)** genus level in mouse pups. In the two-dimensional matrix, the color change reflects the data information, and the color depth indicates the data value. The correlation heatmap analysis is based on Spearman rank correlation. Significant differences are shown by *P <0.05, **P <0.01, ***P <0.001.

To focus on the relationship between succinate and the main gut luminal microbiota in the samples of human neonates and mouse pups, a correlation heatmap based on Spearman rank correlation was used. In fecal samples from human neonates, succinate was positively correlated with *Enterobacteriaceae* and negatively correlated with *Lactobacillaceae* and *Staphylococcaceae* at the family level (*P <0.05*). And we found succinate is negatively correlated with most other species at the family level but not statistically significant (**Figure 2A**). At the genus level, succinate exhibited a significantly positive correlation with *Escherichia-Shigella* and *unclassified_f_Enterobacteriaceae*, and a significantly negative correlation with *Lactobacillus*, *Staphylococcus*, and some *unclassified genera* (*P <0.05*). In addition, we found succinate exhibited a positive correlation with *Enterobacter, Klebsiella* and *Halomonas*, but there was no significant difference (**Figure 2B**). In samples from mouse pup’s models, succinate was significantly positively correlated with *Clostridiaceae*, *Enterococcaceae*, *Lachnospiraceae*, and *Peptostreptococcaceae* but negatively correlated with *Lactobacillaceae* at the family level *(P <0.05)*, however, succinate has no significant correlation with other species at the family level (**Figure 2C**). At the genus level, succinate was negatively correlated with *Lactobacillus*, *Streptococcus* and *norank_f_Muribaculaceae* and positively correlated with *Clostridium_sensu_stricto_1*, *Enterococcus* and *Clostridioides* (*P <*0.05), but not significantly correlation with other species at the genus level (**Figure 2D**). Thus, we conclude that increased intestinal succinate correlates closely with intestinal flora changes.

### Comparison of intestinal flora composition

To evaluate whether the sample size was sufficient for our study, the rarefaction curve was generated. We found that with the increase in the number of Reads Sampled, the rarefaction curve gradually flattened both in human and mice samples, which means that the sample size of our study is sufficient for further analysis (**Figure S1A**, **Figure S2A**).

We first analyzed the fecal flora composition from the two human neonate groups. The alpha diversity analysis was used to explore the community richness and diversity. Compared to the non-NEC group, newborns with NEC showed a markedly decrease in Ace index, but there was no significant different in Shannon index between the two groups (**Figures S1B, C**). At the phylum level, *Proteobacteria* and *Firmicutes* were the dominant phyla in the two groups (**Figure S1D**). Circos plot visualized the distribution of microbial community for each sample at the family level and revealed the different flora composition between the two groups (**Figure S1E**).

Then, we conducted a similar analysis of fecal microbiota compositions in mouse pup models. To visualize the changes in microbiota diversity, we compared the relative abundances of 30 main families in a heat map (**Figure S2B**). As revealed by principal component analysis (PCA) at the family level, the fecal flora composition of control mice was significantly different from that of the other three experimental groups (**Figure S2C**). And the Linear Discriminate Analysis Effect Size (LEfSe) analysis was additionally performed to further identify the most differentially abundant taxa from phylum to genus level in all the groups (**Figures S2D, E**).

**Figure 3:**
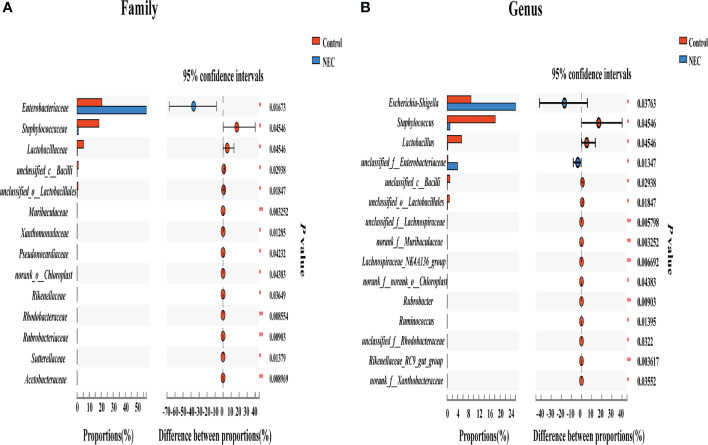
Comparison of fecal flora between NEC patient non-NEC patients. Differences between the control group and the NEC group at the **(A)** family and **(B)** genus levels using the Wilcoxon signed-rank test. Significant differences are shown by *P <0.05, **P <0.01.

**Figure 4:**
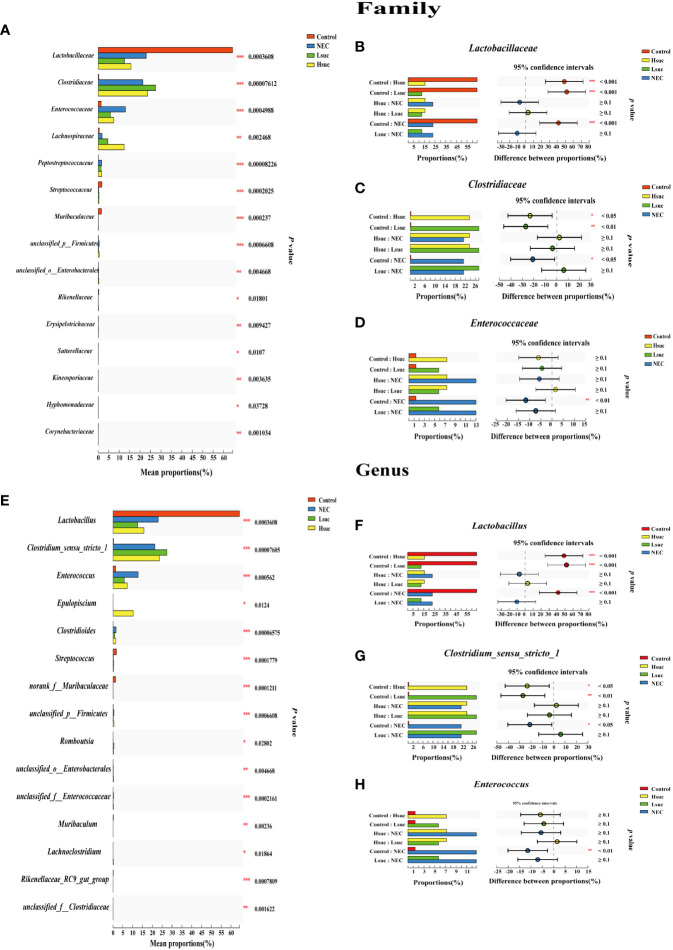
Comparison of community abundance within four independent groups. Differences in flora composition among groups of mice at the **(A–D)** family and **(E–H)** genus levels using the Kruskal – Wallis test with Dunn’s multiple comparison *post hoc* test. Significant differences are shown by *P <0.05, **P <0.01, ***P <0.001.

To investigate differences in the gut flora composition between groups, the major species among the different groups were compared. Our results indicated that human neonates with NEC presented a significantly increased abundance of *Enterobacteriaceae* at the family level and *Escherichia_Shigella* at the genus level but a significantly decreased abundance of *Staphylococcaceae* and *Lactobacillaceae* at the family level and *Staphylococcus*, *Lactobacillus* and some other *unclassified genera* at the genus level compared to those without NEC (**Figures 3A, B**). In mouse samples, *Lactobacillaceae* at the family level and *Lactobacillus* at the genus level were significantly increased, while *Clostridiaceae* and *Enterococcaceae* at the family level and *Clostridium_sensu_stricto_1* and *Enterococcus* at the genus level were significantly decreased in the control group compared to the NEC group. Additionally, there was no significant difference between the succinic acid-intervened groups and the NEC group in terms of species composition at the family and genus levels (**Figures 4A–H**).

### Succinate aggravates the severity of NEC in mice

**Figure 5:**
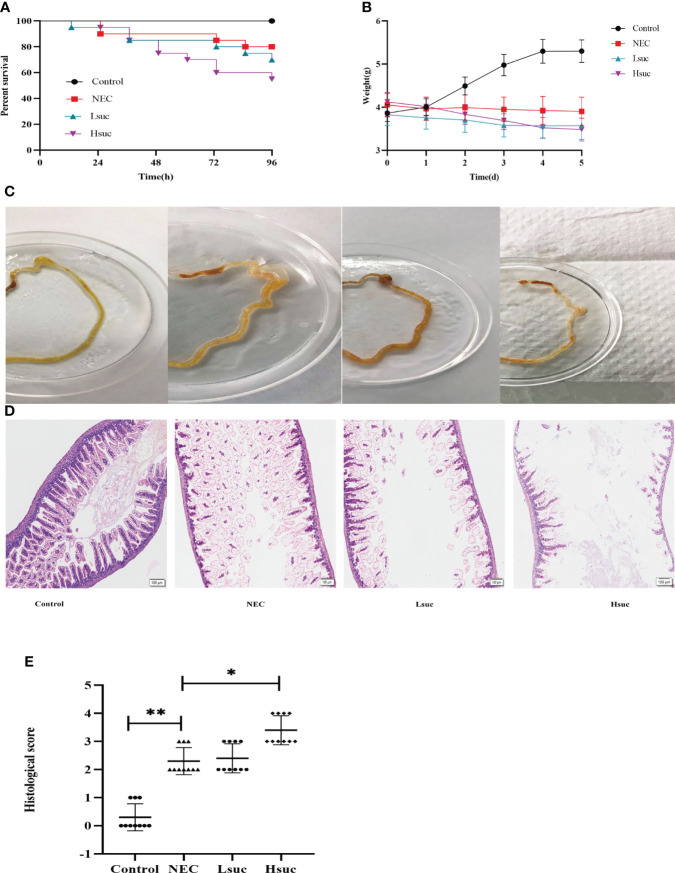
Succinate exacerbates experimental murine necrotizing enterocolitis. Data are from four independent experiments. **(A)** Percent survival in the four groups. Log-rank test *P* value: *P < 0.05. **(B)** Body weight changes in mouse pups in the four groups. Baseline differences in weight were not significant. Two-way ANOVA multiple-comparison method *P* value: *P < 0.05. **(C)** Representative morphological observations of intestinal tissue of each group immediately following excision at the time of the experimental endpoint. **(D)** Representative photomicrographs of terminal ileal sections from each of the four groups. Magnification ×100; scale bars:100 μm. **(E)** Histological scoring of intestinal regions observed in the control, NEC, Lsuc and Hsuc groups. Kruskal – Wallis test P value: *P <0.05, **P <0.01.

To explore the contribution of succinate to necrotizing enterocolitis development, the effects of two different concentrations of exogenous succinic acid on mouse models of NEC were evaluated. During the evaluation of the model mice, the control group exhibited no deaths, in the NEC group, the mortality was 20%, whereas in the Lsuc group and Hsuc group, it was 30% and 45%, respectively (**Figure 5A**). During the modeling period, mouse pups of the Lsuc and Hsuc groups experienced a marked drop in weight, but there was no significant change in weight of the mice with NEC, while the mice from control group showed good weight gain (**Figure 5B**). Naked-eye detection of the intestine segments observed severe dilatation of intestinal lumen and color change of the intestinal wall in the NEC, Lsuc and Hsuc groups, whereas no obvious intestinal damage was observed in the control group (**Figure 5C**). According to histological analysis, the Hsuc group suffered severe intestinal damage and presented significantly higher histological scores than the NEC group (**Figures 5D, E**). Therefore, abnormally high succinate levels can prevent mice from gaining weight, causing intestinal damage, and increasing mortality.

### Succinate-induced disruption of murine intestinal proinflammatory and anti-inflammatory balance

**Figure 6:**
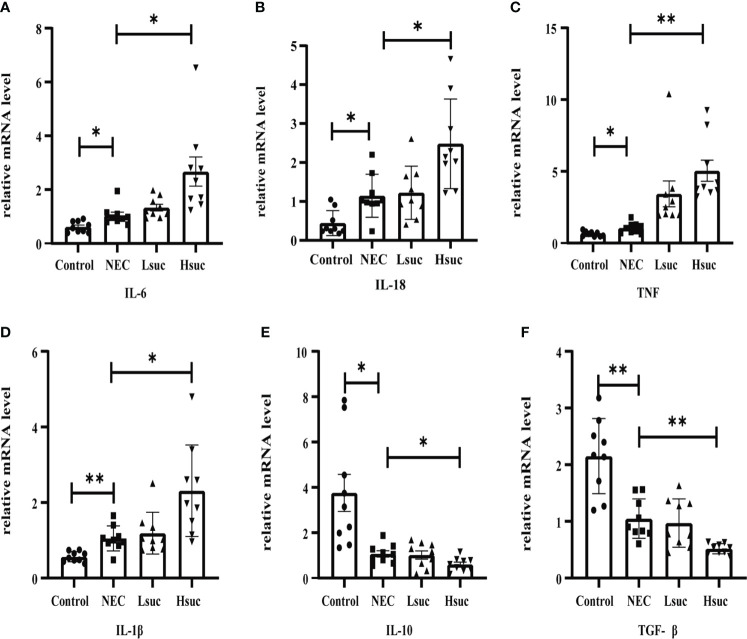
Relative mRNA expression of inflammatory cytokines in the intestines of mice from four independent groups (n=9 per group). **(A–D)** Proinflammatory factors; **(E, F)** anti-inflammatory cytokines. Dots indicate data from individual mice, and data are shown as the means ± SD. Statistical significance was assessed with one-way ANOVA followed by Dunnett’s *post hoc* test. *P < 0.05, **P < 0.01.

To further elucidate the effect of succinate on intestinal inflammation in mice, the mRNA expression levels of pro- and anti-inflammatory cytokines in distal ileum lysates from four independent groups were detected. First, compared with control mice, the levels of proinflammatory factors, including interleukin-1β (IL-1β), IL-6, IL-18, and tumor necrosis factor (TNF), were significantly increased, while the levels of anti-inflammatory cytokines, including IL-10 and transforming growth factor-β (TGF-β) were significantly decreased in murine pups with NEC. Furthermore, the level of the proinflammatory factors IL-6, IL-18, TNF and IL-1β were significantly increased, but those of the anti-inflammatory factors IL-10 and TGF-β were significantly decreased, in the intestinal tissues from the Hsuc group compared with the NEC group. However, the expression levels of anti- and proinflammatory factors were not significantly different between the NEC and Lsuc groups or between the Hsuc and Lsuc groups (**Figures 6A–F**). Thus, elevated luminal succinate levels can drive the proinflammatory response of the intestine, probably in a dose-dependent manner.

### Exogenous succinate upregulates inducible nitric oxide synthase expression while reducing Arginase-1 expression in mice

**Figure 7:**
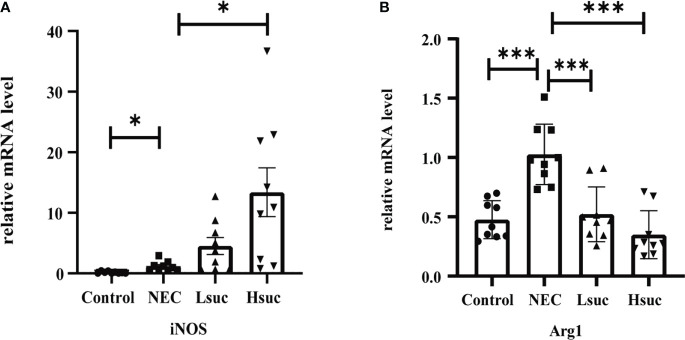
Relative mRNA expression of macrophage markers in the intestines of mice from four independent groups (n=9 per group). **(A)** iNOS, **(B)** Arg1. Dots indicate data from individual mice, and data are shown as the means ± SD. Statistical significance was assessed with one-way ANOVA followed by Dunnett’s *post hoc* test. *P < 0.05, ***P < 0.001.

To preliminarily explore the effect of succinate on macrophage activation, we evaluated the expression of macrophage-specific markers, including inducible nitric oxide synthase (iNOS) and Arginase-1 (Arg1). The NEC group showed significantly higher expression levels of iNOS and Arg1 than the control group. Additionally, the level of the M1 macrophage marker iNOS was significantly increased, but that of the M2 macrophage marker Arg1 was significantly decreased in the intestinal tissues from the Hsuc group compared with the NEC group. However, the expression levels of iNOS and Arg1 were not significantly different between the Hsuc and Lsuc groups (**Figures 7A, B**). Accordingly, certain degree of succinate increase probably alters the status of macrophages.

### Succinate administration enhances succinate receptor 1 and hypoxia-inducible factor 1a protein expression in mice

**Figure 8:**
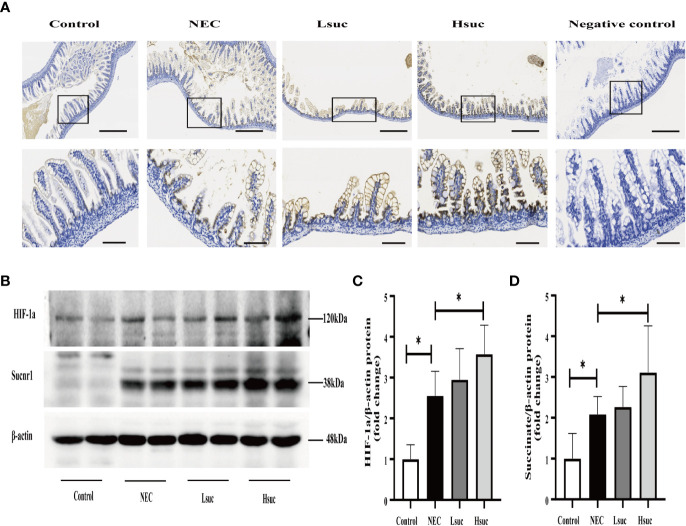
Succinate increases the protein expression of SUCNR1 and HIF-1a in mice. **(A)** Sucnr1 staining intensifies with an increasing of succinate concentration. Representative images of immunohistochemical staining for SUCNR1 in the control group and the NEC, Lsuc and Hsuc groups (n=3 per group). SUCNR1 localizes to the intestinal epithelium and lamina propria cells. Original magnification: ×100 and ×400; scale bar, 100 μm and 20 μm. **(B–D)** A representative western blot and graphs showing SUCNR1 and HIF-1a protein levels in intestine samples from four independent groups (n=7 per group and n=5 per group, respectively). The bars in the graph represent the means ± SD. Statistical significance was assessed with one-way ANOVA followed by Dunnett’s *post hoc* test. *P < 0.05.

To preliminarily explore the role of succinate in the pathogenesis of NEC, we evaluated the protein expression of succinate receptor 1 (SUCNR1) and hypoxia-inducible factor 1a (HIF-1a) in the intestines of mice from four independent experimental groups. We observed that SUCNR1 was localized in the epithelial and lamina propria cells of the mouse small intestine, and the protein staining intensified with an increasing succinate concentration (**Figure 8A**). There were significantly increased protein levels of SUCNR1 and HIF-1a in distal ileum lysates from NEC mice compared to those from control mice. Additionally, mice intervened with 100 mM succinic acid showed significantly higher expression levels of the SUCNR1 and HIF-1a proteins than NEC mice. However, the expression levels of SUCNR1 and HIF-1a were not significantly different between the NEC and Lsuc groups, or between the Hsuc and Lsuc groups (**Figures 8B–D**).

## Discussion

Studies have suggested that gut microbiota dysbiosis and some metabolites play important roles in the occurrence and development of NEC (Moschino et al., 2022). In our study, we found markedly increased concentrations of succinate in fecal samples from human neonates with NEC, and this intriguing finding was validated in murine experiment. Succinate changes were closely related to intestinal flora changes in NEC and abnormally elevated gut luminal succinate levels exacerbate NEC.

Succinate is a cometabolite produced by both hosts and microbes, and it is typically identified at low levels in the intestinal lumen, likely due to its cross-feeding relationships (Connors et al., 2018). Substantial evidence has indicated that succinate concentrations increase under conditions of hypoxia, stress, inflammation, and changes in intestinal microorganisms (Nagao-Kitamoto et al., 2016; Macias-Ceja et al., 2019; Banerjee et al., 2020; Huber-Ruano et al., 2022). It has been widely reported in previous studies that changes in the intestinal flora are closely related to the development of NEC (He et al., 2021; Liu et al., 2022a; Samara et al., 2022). In exploring the pathological mechanism whereby the gut flora affects NEC, some investigators have proven that gut microbiota-derived metabolites, including short-chain fatty acids, DL-lactate, tauroursodeoxycholic acid, and other related metabolites, play important messenger roles in NEC progression or can contribute to its prediction (Li et al., 2019a; Feng et al., 2022; Huang et al., 2022).

Then, we analyzed the relationship between gut luminal succinate and intestinal flora in samples from human neonates and mouse pups. Our data indicated that there were significantly higher proportions of succinate-producing bacteria, including *Enterococcaceae* and *Escherichia_Shigella* (Gradisteanu Pircalabioru et al., 2022), and significantly lower proportions of succinate-consuming bacteria, including *Staphylococcaceae*, *Lactobacillaceae*, *Staphylococcus* and *Lactobacillus* (Shima et al., 2022) in human neonates with NEC than in control human neonates. Among the model mice, there were significantly higher proportions of succinate-producing bacteria, including *Enterococcaceae*, *Clostridiaceae*, *Clostridium_sensu_stricto_1*, and *Enterococcus* (Phuengjayaem et al., 2020) and significantly lower proportions of succinate-consuming bacteria including *Lactobacillaceae* and *Lactobacillus* (Shima et al., 2022) in mice with NEC than in those without NEC. We found that the clinical data were in good agreement with the animal experimental observations. Our findings collectively suggested that gut luminal succinate is very likely derived from the intestinal flora, although a germ-free murine model needs to be constructed to further clarify this hypothesis about NEC.

In our present study, we found that elevated succinate concentrations could prevent NEC mice from gaining weight, damage their intestines, increase their mortality, and simultaneously destroy the balance of anti-inflammatory and proinflammatory factors in the intestine. In recent years, it has been well-established that gut luminal succinate regulates adaptive intestinal remodeling by activating a small intestinal tuft cell-innate lymphoid type-2 cells circuit (Nadjsombati et al., 2018; Schneider et al., 2018). The role of an intestinal gluconeogenic substrate in maintaining intestinal energy metabolism has also been studied (De Vadder et al., 2016). On the other hand, numerous studies point to succinate as a danger signal that can aggravate intestinal mucosal damage and promote the development of inflammation (Fremder et al., 2021; Zhou et al., 2022b). Therefore, we can speculate that succinate may plays its role as a signal molecule depending on the specific cell types or disease states.

We found that SUCNR1 localizes to the epithelium and lamina propria cells of the mouse intestine, our observations are consistent with findings of other studies (Lei et al., 2018; Macias-Ceja et al., 2019). SUCNR1 belongs to the family of G protein-coupled receptors and specifically binds succinate. It has been shown that succinate is important for regulating immune and metabolic functions by activating SUCNR1 (Peruzzotti-Jametti et al., 2018; Mills et al., 2021). In mice, we found that SUCNR1 expression varied with the succinic acid concentration, which has been previously observed (Li et al., 2019b). Some studies have noted that succinate promotes macrophage polarization via the sucnr1- mediated activation of the HIF-1a signaling pathway (Tannahill et al., 2013; Wu et al., 2020). In our research, we observed significantly higher protein expression of HIF-1a in the NEC group than in control normal mice, while the oral administration of succinate also increased the expression of HIF-1a in a dose-dependent manner. Our findings revealed that elevated succinate levels increase the protein expression of SUCNR1 and HIF-1a, suggesting that succinate plays a role in the pathogenesis of NEC, probably by activating the HIF-1a signaling pathway.

We observed significantly increased expression of iNOS and Arg1 in mice with NEC compared to those without NEC. In 100 mM succinic acid – intervened NEC mice, the gene expression of iNOS was significantly increased, while the expression of Arg1 was markedly decreased compared to that in the NEC group. In the innate immune system, macrophages are key regulators of balanced pro- and anti-inflammatory responses. Numerous reports have demonstrated that the aggravation of NEC is closely related to macrophage activation (Wei et al., 2019; Namachivayam et al., 2020). Recently, a study suggested that the uptake of microbe-derived succinate into macrophages, regulated by sodium-coupled citrate transporters, is elevated to maintain the proinflammatory state of the cells (Fremder et al., 2021). Accordingly, our findings suggest that succinate – induced intestinal inflammation may be related to intestinal macrophage activation.

In summary, we found that intestinal succinate levels were increased in NEC, and the increased luminal succinate level was closely related to intestinal flora changes. Abnormally elevated intestinal succinate exacerbates NEC, probably by activating the HIF-1a signaling pathway mediated by SUCNR1, activating macrophages, and disrupting the balance of pro- and anti-inflammatory mediators. Our findings provide new insights into the pathogenesis of NEC and may also help to identify new approaches to treatment for this disease. Nevertheless, the exact mechanism whereby succinate affects the intestine of individuals with NEC needs further elucidation.

## Data availability statement

The datasets presented in this study can be found in online repositories. The names of the repository/repositories and accession number(s) can be found below: https://www.ncbi.nlm.nih.gov/ , PRJNA885748.

## Ethics statement

The studies involving human participants were reviewed and approved by The Ethics Committee of Children’s Hospital of Chongqing Medical University. Written informed consent to participate in this study was provided by the participants’ legal guardian/next of kin. The animal study was reviewed and approved by The Animal Ethics Committee at Chongqing Medical University. Written informed consent was obtained from the minor(s)’ legal guardian/next of kin, for the publication of any potentially identifiable images or data included in this article.

## Author contributions

All the authors made substantial contributions to the study. T-TD, X-CL and X-LY collected the clinical data and worked on basic sample processing. X-CL, T-TD, XG, J-LY and X-LY collected the fecal samples. QA, J-LY, Y-NZ, and X-LY conceived and conducted the experiments and analyzed the data. X-LY wrote the manuscript. LB and L-QL contributed to conceptualization, funding acquisition, and supervision. LB and L-QL contributed to the critical revision and final approval of the manuscript. X-LY, L-QL and LB provided the final approval of the manuscript. All authors contributed to the article and approved the submitted version.

## Funding

This work was supported by the Natural Science Foundation of Chongqing municipality (cstc2021jcyj-msxmX0063); the Joint Medical Research Project of Chongqing Science and Technology Commission (2021MSXM206,2022MSXM039) and the Project of Nestle Health Science (KY210030, China).

## Conflict of interest

The authors declare that the research was conducted in the absence of any commercial or financial relationships that could be construed as a potential conflict of interest.

## Publisher’s note

All claims expressed in this article are solely those of the authors and do not necessarily represent those of their affiliated organizations, or those of the publisher, the editors and the reviewers. Any product that may be evaluated in this article, or claim that may be made by its manufacturer, is not guaranteed or endorsed by the publisher.
