# Supplementary material for: Succinate aggravates intestinal injury in mice with necrotizing enterocolitis
Source: Front Cell Infect Microbiol. 2022 Nov 28;12:1064462. doi: 10.3389/fcimb.2022.1064462 (PMC9742382; doi:10.3389/fcimb.2022.1064462)
Supplement: Supplementary file 1 [file DataSheet_1.docx]

Supplementary Material


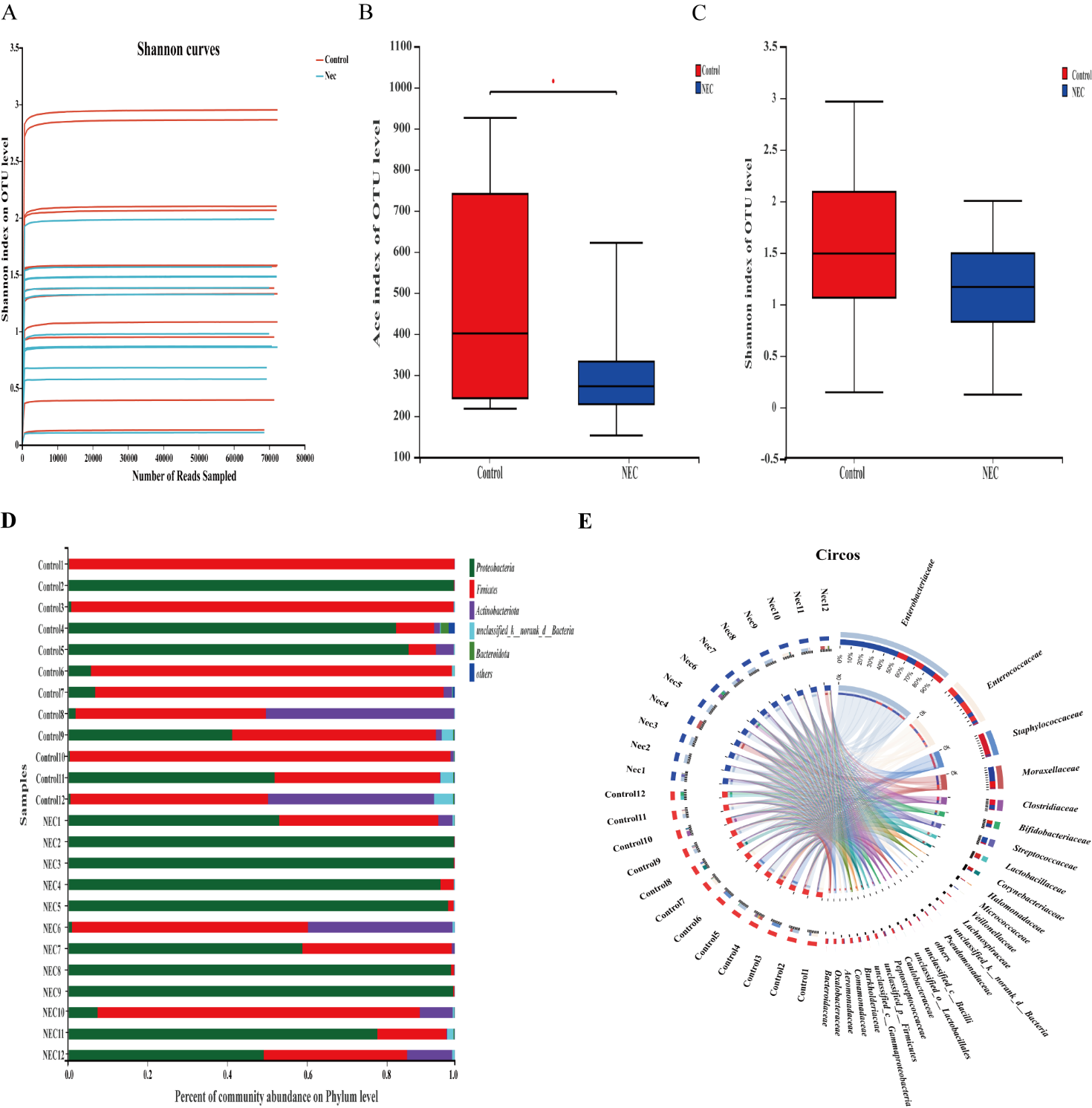


**Figure S1.** Composition of the fecal flora in NEC neonates and non-NEC neonates. (**A**) The rarefaction curve was generated to evaluate the data richness in human neonate samples. **(B** and **C**) Ace and Shannon alpha diversity of the fecal flora based on 16S profiling. Boxes indicate median, first, third quantiles, and whiskers show the range of values from minimum to maximum. Statistical analysis was performed with Mann–Whitney rank-sum test, significant difference is shown by **P* < 0.05. (**D**) Relative community abundance of each sample from the two groups at the phylum level. (**E**) Distribution of microbial community for each sample at the family level, visualized by Circos.


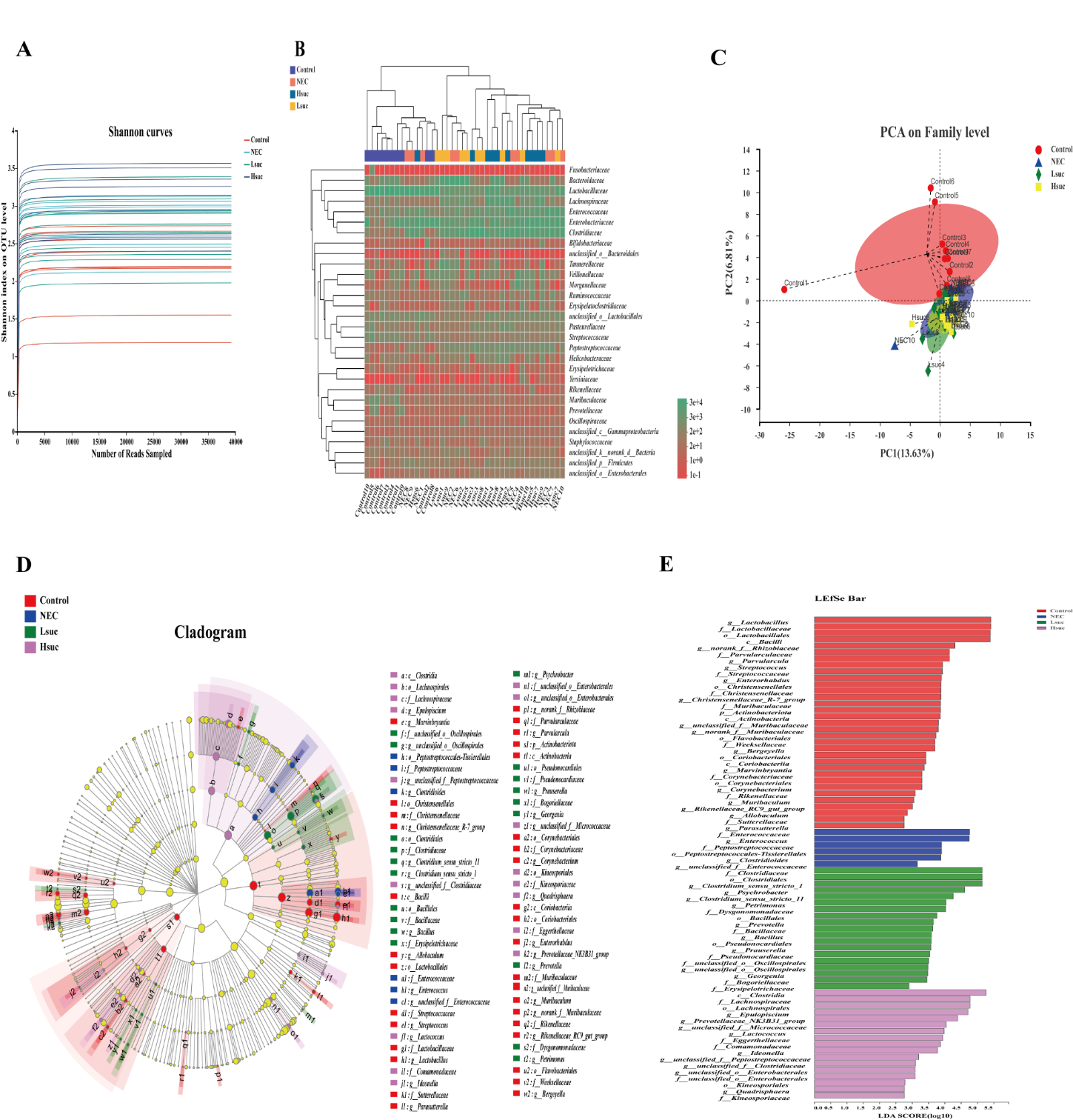


**Figure S2.** Composition of the fecal flora in different mouse groups. (**A**) The rarefaction curve was generated to evaluate the OTU richness in murine samples. (**B**) The bacterial taxonomic heatmap in 30 main families of the gut microbiota. (**C**)The principal component analysis (PCA) at family level revealed that fecal flora composition of the control group was significantly different from the other three experimental groups. (**D** and **E**) Linear discriminate analysis effect size (LEfSe) was performed to screen species with significant differences from phylum to genus level in four groups, the threshold of LDA score was 2.0.
